# Susceptibility and transmission of mpox virus infection in brown rats (Rattus norvegicus)

**DOI:** 10.1099/jgv.0.002125

**Published:** 2025-07-01

**Authors:** Lucy Crossley, Stephen Findlay-Wilson, Linda Easterbrook, Emma Kennedy, Francisco J. Salguero, Kim Mackay, Victoria Graham, Susan Fotheringham, Stuart Dowall

**Affiliations:** 1 UK Health Security Agency (UKHSA), Porton Down, Salisbury, Wiltshire, SP4 0JG, UK

**Keywords:** emerging infectious diseases, monkeypox (mpox) virus, poxvirus, viruses

## Abstract

*A corrigendum of this article has been published full details can be found at* *https://doi.org/10.1099/jgv.0.002162**.*

Mpox (formerly known as monkeypox) virus (MPXV) is the zoonotic pathogen of mpox disease in humans. Its increasing emergence outside of its endemic area has heightened the importance of investigating the virus’ prevalence and maintenance in sylvatic reservoirs. The common brown rat (*Rattus norvegicus*) can inhabit almost anywhere in the UK, posing a threat to zoonotic transmission to humans. Two independent studies were carried out; the first investigated the susceptibility of brown rats to MPXV infection with a clade IIb mpox strain via two challenge routes: intranasal and intradermal. The second study considered the transmission of MPXV between challenged and naïve brown rats. All animals were asymptomatic to mpox disease, although enzyme-linked immunosorbent assay (ELISA) confirmed subclinical infection in challenge groups. In the susceptibility study, reverse transcription PCR (RT-PCR) detected mpox DNA in the lung tissue and throat swabs within the intranasally inoculated group, in addition to viable virus observed from the intranasal throat swabs. In contrast, no virus was detected in either tissues or swabs in the intradermally inoculated group or control group. RT-PCR results from the transmission study detected mpox DNA in tissues and throat swabs taken from challenged animals. Viable virus was observed from tissues and swabs of intranasally challenged animals with infectious titres of ~10^2^–10^4^ TCID_50_ per millilitre. ELISA assays in the transmission study showed replicable results compared to the first susceptibility study in directly challenged animals alongside evidence of seroconversion in co-housed naïve animals. In conclusion, brown rats are susceptible to MPXV infection, as they have been demonstrated to maintain viable virus in the absence of clinical signs. Viral transmission of MPXV from infected rats to naïve rats was not observed by RT-PCR, although naïve rats did show antibody responses when exposed to infected rats indicating exposure to virus.

## Introduction

Monkeypox (mpox) is an emerging zoonotic disease caused by the rodent-borne pathogen mpox virus (MPXV), an *Orthopoxvirus* belonging to the *Poxviridae* family [1]. The growing prevalence of MPXV is fast becoming a cause for concern due to the expansion of human infections outside of its endemic area. Although mpox disease has been well characterized in humans, its maintenance in animal populations of sylvatic reservoirs is not well known [2]. Previous work has been undertaken in African squirrels and dormice, as well as non-African species including prairie dogs, ground squirrels and inbred mice [3]. More recently, investigations have been undertaken in African pouched rats (*Cricetomys gambianus*), which are involved in the transmission and maintenance of MPXV in Africa [4] and associated with the importation of MPXV into the USA during 2003 [5]. Laboratory challenge studies demonstrated that viable virus was detectable between 3 and 27 days post-infection (dpi) [2]. Whilst previously endemic to Africa, these rats have been introduced to Grassy Key, Florida, due to activities associated with the exotic pet trade. Another species of rodent, white rats (*Rattus norvegicus*), was assessed for susceptibility in the 1970s where only newborn (1–3 days) animals showed clinical signs [6].

The brown rat (*R. norvegicus*), also known as the common rat, is a widespread species present all over the globe alongside humans [7]. The brown rat can be found almost anywhere in the UK, in any habitat. A systematic review revealed that 48 different infectious agents, all with zoonotic potential, could be carried by commensal brown rats [8].

Whilst we have found no evidence of brown rats being assessed for MPXV susceptibility, this work will complement previous studies on cowpox virus (CPXV), one of the four species of the genus *Orthopoxvirus*, family *Poxviridae*, which are pathogenic to humans; the others being MPXV, variola and vaccinia. Wistar (*R. norvegicus*) and fancy (*R. norvegicus domestica*) laboratory rat strains have been shown to be highly susceptible to CPXV infection [9,10]. Brown rats have been shown to be susceptible to CPXV [11] in addition to the closely related ratpox virus [12]. Indeed, rats (often referred to as brown rats) have been known to transmit CPXV to other animal species including monkeys [13] and elephants [14], along with humans via wild animals [15] and through pets [16,17]. Whilst rats may be a reservoir, human transmission of CPXV may also be associated with domestic cats [18], hypothesized to be infected via direct contact during hunting of wild rodents.

Precedents exist for introductions, whether accidental or intentional, of poxviruses into novel host systems, including myxoma virus in Australia and Europe [19,20] and vaccinia virus in South America [21,22]. Given the current global expansion of MPXV cases [23], there is an increased chance of brown rats being exposed to this virus. This may be direct, as the brown rat has been selectively bred to produce fancy rats kept as pets (*R. norvegicus domestica*), or it may be via exposure to virus in, for example, sewage systems – especially as viable virus has been found in faecal and urine samples from prairie dogs, suggesting the potential for fomite transmission [5]. The multi-country MPXV outbreak in 2022 resulted in the World Health Organization (WHO) declaring it a public health emergency of international concern (PHEIC) [24]. During this outbreak, MPXV exposure was associated with sexual activity [25], with clinical presentation involving areas around the human excretory system, with up to 31.43% of cases exhibiting genital and anal lesions [26]. Thus, there is a strong likelihood that infectious virus may enter the wastewater system, thus providing an opportunity to initiate infection in brown rats. Identifying MPXV sylvatic reservoirs is important for the identification of public health risks and for the improvement of surveillance and implementation of infection control measures.

## Methods

### Animals

To determine susceptibility to infection, 12 female brown rats (*R. norvegicus*), aged 4–5 weeks, were obtained from a UK Home Office-accredited supplier (Envigo, UK). The animals were assigned into groups determined by challenge route: intranasal (*n*=5), intradermal (*n*=5) and unchallenged negative controls (*n*=2).

A second study assessed transmissibility between intranasally challenged and naïve rats alongside a scheduled cull to assess local responses at the peak infection timepoint. Nine male and nine female brown rats, aged 4–5 weeks, were obtained from a UK Home Office-accredited supplier (Envigo, UK). The animals were assigned into two groups with an equal sex allocation: a transmission group (*n=*12), where one intranasally challenged animal was housed with one naïve animal, and a scheduled cull group (*n=*6) taken at peak infection and not co-housed with a naïve cagemate.

### Virus

MPXV was isolated from a human case identified during the 2022 MPXV outbreak in the UK (sequence accession no. OP394229) and made available through the National Collection of Pathogenic Viruses (product number 2206091v) and European Virus Archive (ref: 004 V-04757). The virus was propagated for two passages in Vero E6 cells (accession number 85020205; European Collection of Cell Cultures, UK) in Minimum Essential Media-ɑ supplemented with 2% FBS, 25 mM HEPES, 1× non-essential amino acids and 1× antibiotic–antimycotic solution (Gibco, UK).

### Virus challenge

The infectious titre of the virus was determined by TCID_50_ in Vero E6 cells. On the day of challenge, rats received 4×10^5^ TCID_50_ MPXV inoculation in a 100 µl suspension. For the susceptibility study, those in the intradermal group received this dose over two sites, 50 µl medial and 50 µl dorsal, and those in the intranasal group had 50 µl delivered to each nare. Animals within the control group were left unchallenged. For the transmissibility study, challenged animals were inoculated intranasally receiving 50 µl in each nare and housed alongside a naïve sex-matched cagemate.

### Clinical observations and sample collection

Following infection, all animals were monitored twice daily for clinical signs of disease. Animals were weighed, and temperatures were measured by indwelling temperature chips for up to 21 dpi by experienced husbandry and animal welfare staff. On 0, 2, 4, 6, 8, 10, 13, 16 and 19 dpi, throat and rectal swabs were collected from each animal whilst under anaesthesia. A dry flocked mini-tip swab (product MW002NF, MWE, Corsham, UK) was used for sampling and added to 1 ml Virocult universal transport medium (product MW951T, MWE, Corsham, UK). At necropsy, the following tissues were collected: liver, spleen, kidney, brain, lung and intestine. A small sample (~5 mm^3^) was added to a PreCellys tube containing ceramic beads (CK28R, Stretton Scientific, UK), and the remainder was added to a histology pot containing 10% formalin fixative. For the blood, 100 µl was collected into an RNAprotect tube (Qiagen, UK) and the remainder into a serum separation tube (Becton Dickinson, UK) for sera preparation. Tubes were stored at −80 °C prior to processing.

### Real-time RT-PCR assay

Swab samples were processed by adding 140 µl sample to 560 µl AVL buffer (Qiagen, UK) followed by 560 µl of 100% ethanol after 10 min to complete the inactivation process. Tissue samples were weighed before the addition of 500 µl PBS to each sample tube. The samples were disrupted on an automated homogenizer (PreCellys 24). For tissue sample inactivation, 200 µl sample was added to 600 µl RLT buffer, followed by 600 µl of 70% ethanol after 10 min. DNA from swab and tissue samples was extracted using the Bio-Sprint All-For-One Vet Kit (Indical, UK) on the KingFisher Flex system platform (ThermoFisher, UK), following the manufacturer’s guidelines. DNA was eluted in 100 µl AVE buffer (Qiagen, UK). The primers and probe set used were developed by the Centres for Disease Control and Prevention targeting the TNF receptor gene (OPG002) for generic MPXV detection [27]. For each reaction, primer and probe concentrations were made to 900 and 250 nM, respectively. Samples were analysed by reverse transcription PCR (RT-PCR) using the TaqMan Fast Virus Step-1 Master Mix RT-PCR kit (Thermo Fisher, UK) and run with the fast-cycling mode: reverse transcription at 50 °C for 5 min, denaturation at 95 °C for 20 s, followed by 45 and 40 cycles, for the susceptibility and transmissibility study, respectively, at 95 °C for 3 s and 60 °C for 30 s with a cool step of 40 °C for 30 s. MPXV DNA was used as a positive DNA control in conjunction with non-template negative controls.

### Virus infectivity in tissue culture

RT-PCR-positive samples from day 4 throat swabs and lung tissue from the susceptibility study were assessed in tissue culture to evaluate the presence of viable virus. The remaining swab solution or tissue homogenate of these samples was added to Vero E6 cells and incubated at 37 °C with 5% CO_2_ for up to 7 days, with observation for the presence of virus observed microscopically for cytopathic effect (CPE). To evaluate virus infectivity of RT-PCR-positive samples from the transmission study, throat swabs and tissues that were RT-PCR positive on day 6 dpi were titrated by TCID_50_ on Vero E6 cells to determine live viral titres.

### Histopathological analysis

Formalin-fixed tissue samples were processed routinely into paraffin wax. Four-micrometre sections were cut and stained with haematoxylin and eosin (H and E). In addition, tissue sections were stained using the *in situ* hybridization (ISH) RNAscope technique to identify MPXV nucleic acid. Briefly, slides were pre-treated with hydrogen peroxide for 10 min (room temperature), target retrieval for 15 min (98–101 °C) and protease plus for 30 min (40 °C) (Advanced Cell Diagnostics, USA). A V-mpox-specific probe (Cat No. 534678, Advanced Cell Diagnostics) was incubated with the tissues for 2 h at 40 °C. Amplification of the signal was carried out following the RNAscope protocol using the RNAscope 2.5 HD Detection Kit – Red (Advanced Cell Diagnostics). Each ISH run included appropriate negative and positive controls. All slides were digitally scanned using a Hamamatsu S360 digital slide scanner (Hamamatsu Photonics K.K., Shizuoka, Japan) and evaluated subjectively by a qualified pathologist using ndp.view2 software (Hamamatsu Photonics K.K., v2.8.24). The pathologist was blinded to treatment and group details, and the slides were randomized prior to examination in order to prevent bias (blind evaluation). Histopathology and the ISH technique were carried out in an ISO9001 : 2015 and good laboratory practice-compliant laboratory.

### Enzyme-linked immunosorbent assay

An enzyme-linked immunosorbent assay (ELISA) was used to evaluate the IgG response to MPXV in both the susceptibility and transmission studies. A pool of recombinant MPXV antigens A35, B2, B6, VACV B5 and E8 (Native Antigen Company, UK) was diluted in sodium bicarbonate coating buffer (Sigma, UK) at a concentration of 0.1 µg ml^−1^. A volume of 100 µl of 0.1 µg ml^−1^ antigen in coating buffer was added to 94 wells of a 96-well plate, and sodium bicarbonate buffer coating was added to the remaining two wells for a non-Ag coated control. Plates were covered with plate sealers and incubated at 2–8 °C overnight. The plates were washed three times with 200 µl of PBS containing 0.05% Tween 20 (PBS-T) (Sigma-Aldrich, UK) before blocking with 200 µl per well of 5% skimmed milk powder (Merck, Millipore, UK) in PBS-T and incubating for 1 h at 37 °C. Blocking buffer was removed from the wells, and samples were plated from 1 : 50 to 1 : 781,250 in a 1 : 5 dilution series. Pre-challenge rat sera were used as a negative control sample. Plates were sealed with a plate sealer and incubated at 37 °C. After 1 h incubation, plates were washed three times before the addition of 100 µl per well of 1 : 5,000 anti-rat IgG HRP conjugate (Invitrogen, UK) and incubated at 37 °C for 1 h. Plates were washed three times before the addition of 100 µl per well of TMB substrate and incubated at room temperature for 20–30 min. The reaction was stopped with 50 µl per well of stop solution. OD was measured at 450 nm with a SpectraMax spectrophotometer within 20 min of assay completion.

### Statistical analysis

Statistical analysis was carried out using Minitab software (version 19.1.0.1). A non-parametric Mann–Whitney U test was performed to conduct comparisons between groups to determine statistical significance. A statistical significance level of *P*≤0.05 was considered statistically significant.

## Results

### Clinical signs

**Fig. 1.**
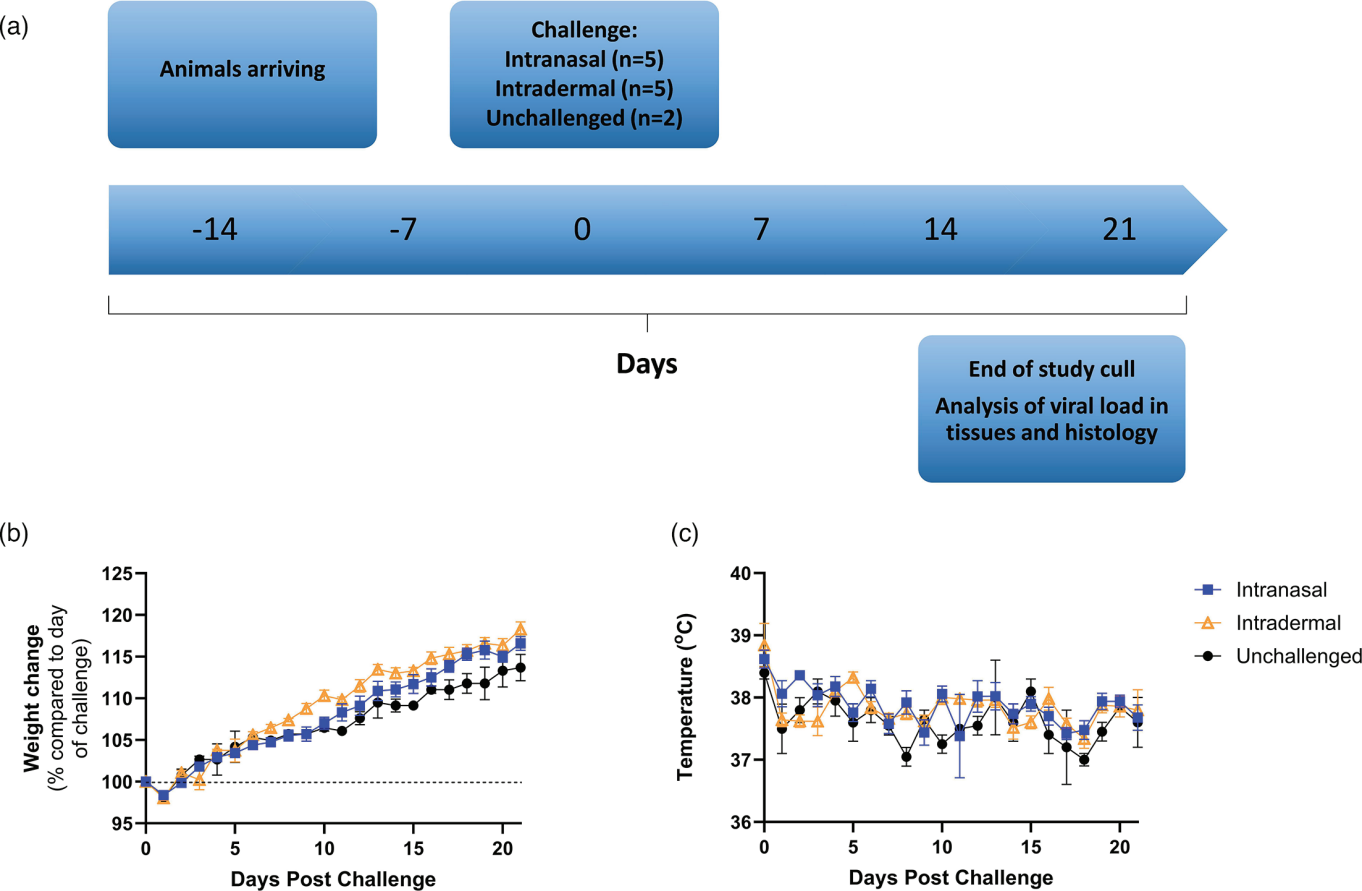
Susceptibility study outline and clinical observations. Brown rats were inoculated with 4×10^5^ TCID_50_ MPXV through intranasal (*n*=5) or intradermal (*n*=5) challenge route and compared to unchallenged control animals (*n*=2). All animals continued to be monitored for 21 days after challenge and did not show signs of any illness. (**a**) Schematic outline of susceptibility study plan. (**b**) Body weight change shown as percentage compared to day of challenge. (**c**) Temperature measurements. Symbols show mean values with error bars denoting standard error.

To assess susceptibility, two groups of female brown rats were challenged with MPXV: one group via the intranasal route and a second group via intradermal challenge (Fig. 1a). All animals survived the scheduled length of the study (21 dpi) and showed no clinical signs of disease, including the absence of visible external lesions. Animals in both challenge groups showed a gradual increase in weight over time, indicating that they remained healthy throughout the study (Fig. 1b); this was supported with temperatures remaining consistent throughout the study (Fig. 1c). No significant differences were found between challenge groups and the control.

**Fig. 2.**
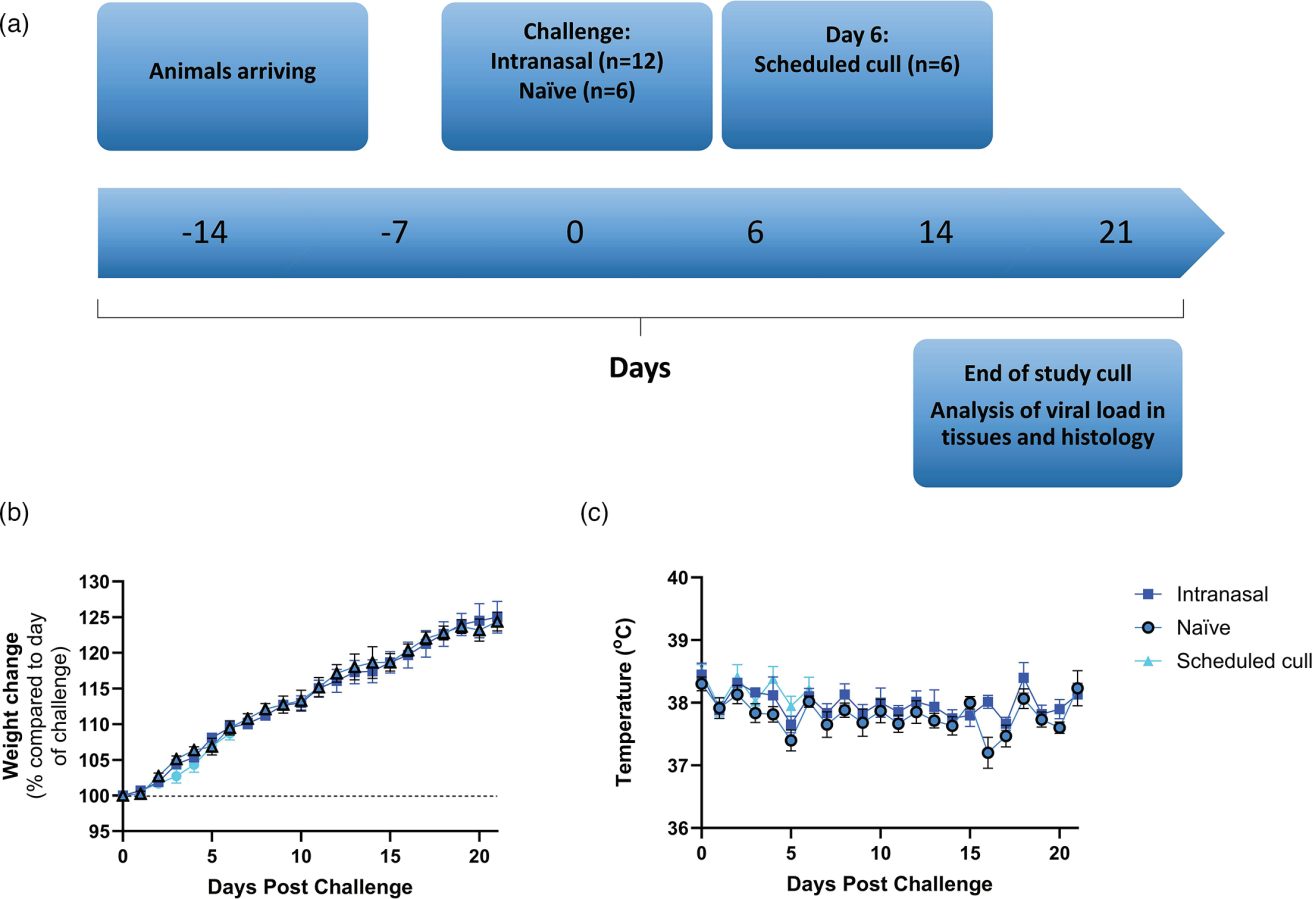
Transmission and reproducibility study outline and clinical observations. Brown rats were inoculated with 4×10^5^ TCID_50_ MPXV through the intranasal (*n*=12) challenge route. Single intranasally challenged rats (*n*=6) were co-housed with a single naïve cagemate (*n*=6), and all remaining intranasally challenged rats (*n*=6) were scheduled for cull at day 6 post-challenge for histopathological analysis. All animals continued to be monitored for 21 days after challenge and did not show signs of any illness. (**a**) Schematic outline of the study plan. (**b**) Body weight change shown as percentage compared to day of challenge. (**c**) Temperature measurements. Symbols show mean values with error bars denoting standard error.

To assess transmission of MPXV, male and female rats were assigned into two groups; one group housed each intranasally challenged animal alongside a naïve cagemate (*n=*12), and a second group planned a scheduled cull of intranasally challenged animals on the day of peak infection (*n=*6). All animals in the transmission study survived to the end of the scheduled study (21 dpi) with no clinical signs of disease. However, five animals developed external scabbing on the skin from 16 dpi observed around the head from both challenged and naïve rats consistent with signs of overgrooming and not mpox disease. All animals showed an increase in weight over the scheduled length of the study and remained healthy throughout (Fig. 2b). Daily temperature observations also remained consistent (Fig. 2c). No significant differences were found between challenged and naïve animals.

### Viral load analysis and viral titration

**Fig. 3.**
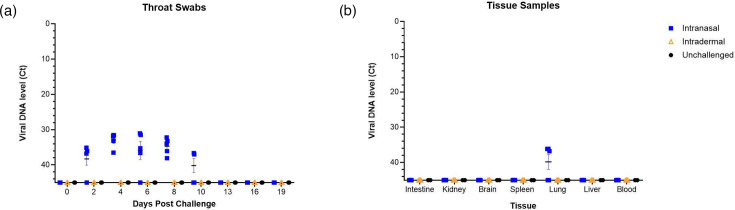
Viral DNA levels from the susceptibility study in the throat swabs taken at regular intervals post-challenge and in tissue samples collected at necropsy. Brown rats were inoculated with 4×10^5^ TCID_50_ MPXV by intranasal (*n*=5) or intradermal (*n*=5) route, and control animals remained unchallenged (*n*=2). Throat and rectal swabs were taken at 0, 2, 4, 6, 8, 10, 13, 16 and 19 dpi, and a tissue sample was taken at necropsy (21 dpi) and tested for the presence of MPXV DNA by RT-PCR. Results showed as Ct values. (**a**) Throat swabs. (**b**) Tissue samples collected at 21 dpi. (**a, b**) Symbol shows mean with error bars denoting standard error.

An RT-PCR assay targeting the OPG002 gene was used, which previously had detected a recently circulating UK strain of MPXV IIb from clinical samples. In the first independent study for susceptibility, viral DNA was detected in the throat swabs and lung tissues of intranasally infected animals (Fig. 3a, b, respectively). Increasing viral DNA was observed from 2 dpi and peak viral DNA levels on 4–8 dpi but was undetectable at 13 dpi. In contrast, no MPXV DNA was observed in the swabs taken from animals in the intradermally challenged and control groups. Additionally, there was no detection of virus in the rectal swabs from either challenge route. MPXV DNA was detected in three lung samples taken at the end of the study from animals in the intranasally challenged group with Ct values of 36.1, 36.8 and 37.1. No other tissues were positive for MPXV DNA.

**Fig. 4.**
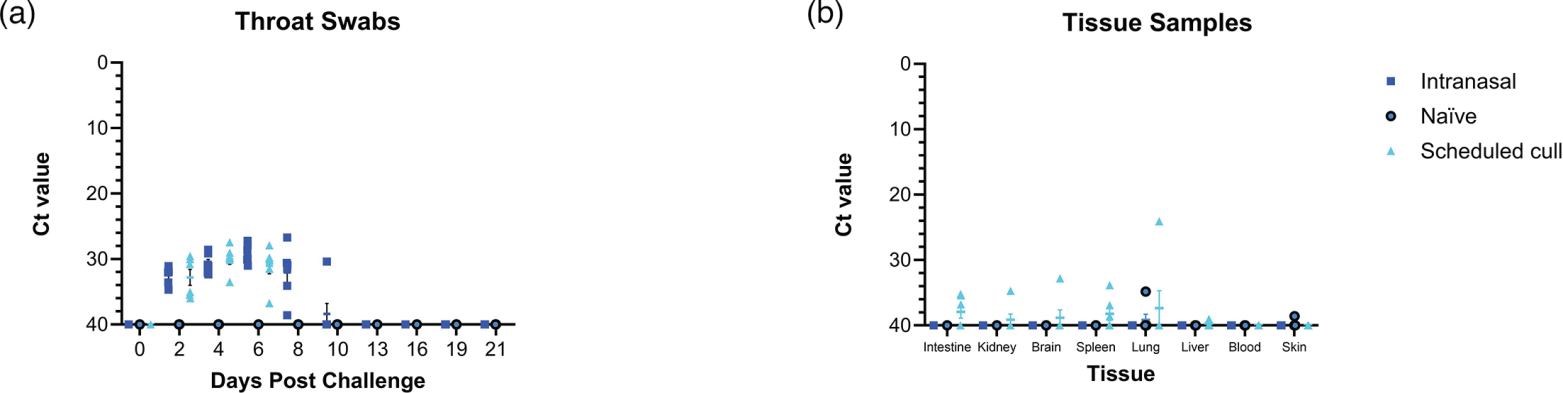
Viral DNA levels from the transmission study in the throat swabs taken at regular intervals during the study and in tissues taken at necropsy. Brown rats were inoculated with 4×10^5^ TCID_50_ MPXV by the intranasal (*n*=12) route, and naïve cagemates (*n*=6) remained unchallenged. Throat swabs were taken at 0, 2, 4, 6, 8, 10, 13, 16 and 19 dpi from all animals. Tissue samples were taken from scheduled cull animals at 6 dpi, and all remaining animals at necropsy (21 dpi). Throat swabs and tissue samples were tested for the presence of MPXV DNA by RT-PCR. Results showed as Ct values. (**a**) Throat swabs. (**b**) Tissue samples collected at 6 dpi (*n*=6) and 21 dpi (*n*=12). Symbol shows means with error bars denoting standard error.

In the transmission study, viral DNA was detected in throat swabs of challenged animals, whereas, in contrast, no viral DNA was detected in throat swabs of naïve animals throughout the study (Fig. 4a). Viral DNA in the throat swabs increased from 2 dpi for all challenged animals with peak infection at 6 dpi before viral DNA was undetectable by 13 dpi. MPXV DNA was detected in tissues taken from animals at 6 dpi, in the intestine (Ct 35.2 and 36.8), kidney (Ct 34.7), brain (Ct 32.8), spleen (Ct 33.8) and lung (Ct 24.0) (Fig. 4b). Two challenged animals taken at 6 dpi had one replicate above the threshold for spleen at Ct 36.9 and 38.6. One lung tissue sample from a naïve animal in the transmission study detected positive for MPXV DNA at 21 dpi with a Ct 34.8, and one naïve animal showed one replicate above threshold in a skin sample taken from a scab formed during the study with a Ct 38.6 (Fig. 4b). At time of necropsy, no skin samples were taken at the site of intradermal infection for MPXV RT-PCR. No tissues from intranasally challenged animals taken at 21 dpi were positive for MPXV DNA.

All throat swabs taken from challenged animals at day 6 dpi in the transmission study were positive for MPXV DNA, replicating the peak of infection observed in the first study for susceptibility of virus. Infectious virus was detected by TCID_50_ from throat swabs of challenged animals taken at 6 dpi and tissues that were positive by PCR (Table 1). Titres of infectious virus were obtained in 11 out of 12 throat swabs of challenged animals. Throat swabs with Ct values as low as Ct 27 obtained viral titres as high as 2.00×10^4^ TCID_50_ per millilitre. Titres of 1.12×10^2^ TCID_50_ per millilitre were obtained and corresponded with higher Ct values (Ct 30.10 and 31.5). Tissues from four challenged animals were positive for live virus by TCID_50_, and titres were obtained in the brain (2.00×10^4^ TCID_50_ per millilitre), kidney (1.12×10^3^ TCID_50_ per millilitre), lung (3.56×10^4^ TCID_50_ per millilitre) and spleen (6.32×10^3^ TCID_50_ per millilitre). Viral titres were unable to be obtained from one tissue and one swab of challenged animals that were detected above Ct 35 by PCR. One lung tissue of a naïve animal that was determined positive by RT-PCR was negative for live virus by TCID_50_.

**Table 1.** Independent study: transmission. Viable virus replication was detected by TCID_50_ assay from RT-PCR-positive throat swab samples at peak infection (6 dpi) and positive tissue samples from brown rats infected by intranasal challenge with MPXV

Intranasal	Sample type	Ct	TCID_50_ per millilitre
	Swab	30.10	6.32×10^2^
	Swab	29.90	3.56×10^3^
	Swab	27.20	2.00×10^4^
	Swab	27.90	1.12×10^4^
	Swab	31.00	1.12×10^3^
	Swab	31.50	1.12×10^2^
	Swab	28.60	2.00×10^3^
	Swab	36.70	–
	Swab	29.70	1.12×10^3^
	Swab	30.10	2.00×10^3^
	Swab	27.90	1.12×10^3^
	Swab	29.90	6.32×10^3^
	Tissue (brain)	32.80	2.00×10^4^
	Tissue (intestine)	35.20	–
	Tissue (kidney)	34.72	1.12×10^3^
	Tissue (lung)	24.07	3.56×10^4^
	Tissue (spleen)	33.84	6.32×10^3^
**Naïve**			
	Tissue (lung)	34.81	–

### Histopathology

**Fig. 5.**
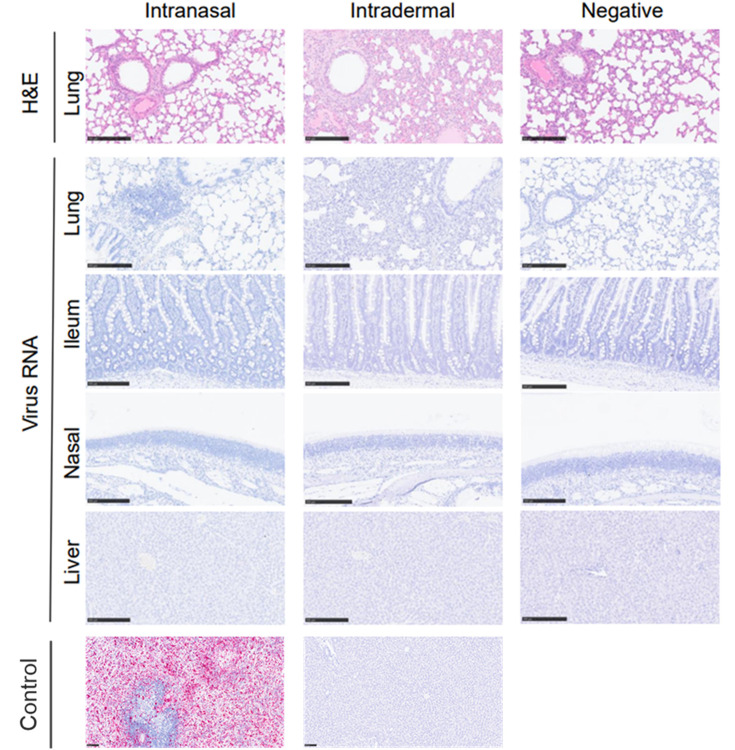
Representative histopathological images of rats challenged with MPXV. H and E staining of the lung and ISH (RNAscope) images of the lung, ileum, nasal cavity and liver. Bar=250 µm. The positive and negative controls include a spleen from an MPXV-positive experimental non-human primate and liver from a naïve animal.

No significant histopathological lesions were observed in any of the examined tissues in the first susceptibility study. Background changes such as necrosis of the tip of villi in the small intestine were occasionally observed. Moreover, no positive staining was observed in any ISH section (Fig. 5).

**Fig. 6.**
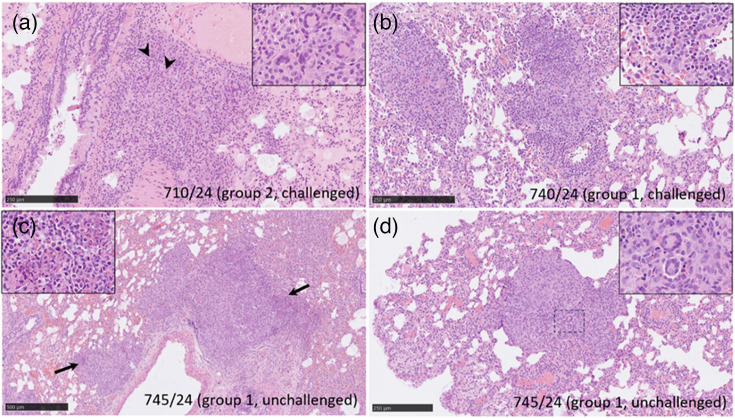
Representative images (H and E) of lung histopathology. (**a**) Section from sample 710/24 (group 2, challenged) showing an area of granulomatous pneumonia with abundant macrophages and giant cells (arrowheads and inset) infiltrating the alveolar septa. (**b**) Section from sample 740/24 (group 1, challenged) showing multifocal granulomatous pneumonia with a high number of macrophages and polymorphonuclear cells (inset) infiltrating the alveolar septa. (**c**) Section from sample 745/24 (group 1, unchallenged) showing multifocal granulomatous pneumonia with a high number of macrophages and polymorphonuclear cells (inset). (**d**) Section from the same animal that (c) shows an area of granulomatous pneumonia with a high number of macrophages, polymorphonuclear cells, lymphocytes and a few giant cells (inset) infiltrating the alveolar septa. Scale bars (a, b, d) 250 µm and (c) 500 µm.

**Fig. 7.**
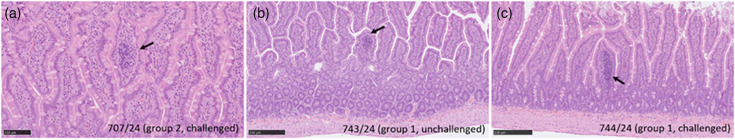
Representative images (H and E) of small intestine. (**a**) Section from sample 707/24 (group 2, challenged) showing an inflammatory infiltrate with few mononuclear cells in a villus (arrow). (**b**) Section from sample 743/24 (group 1, unchallenged) showing an inflammatory infiltrate with few mononuclear cells in a villus (arrow). (**c**) Section from sample 744/24 (group 1, challenged) showing an inflammatory infiltrate with few mononuclear cells in a villus (arrow). Scale bar (b, c) 250 µm and (a) 100 µm.

**Fig. 8.**
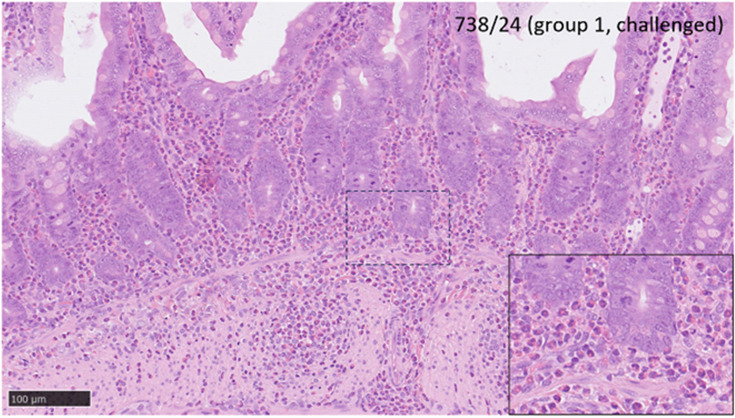
Representative image (H and E) from sample 738/24 (group 1, challenged) of intestine. Abundant polymorphonuclear cells infiltrate the lamina propria and muscularis layer of the mucosa. Inset shows higher magnification. Scale bar 100 µm.

Histopathological and RNAscope ISH techniques observed no specific lesions related to MPXV infection in any studied animal from the second independent study assessing transmission. Mild to moderate histopathological changes consisting of multifocal areas of granulomatous pneumonia, compatible with aspiration pneumonia, were observed in the lungs from challenged and unchallenged animals (Fig. 6). Histopathological changes observed minimal to mild enteritis in the small intestines characterized by the presence of mononuclear cells from challenged and unchallenged animals (Fig. 7). One challenged animal in the transmission study observed moderate infiltration of polymorphonuclear cells in the lamina propria and *muscularis* layer of the small intestine (Fig. 8). No presence of MPXV DNA was observed associated with these lesions or any other tissue from challenged or unchallenged animals.

### ELISA

**Fig. 9.**
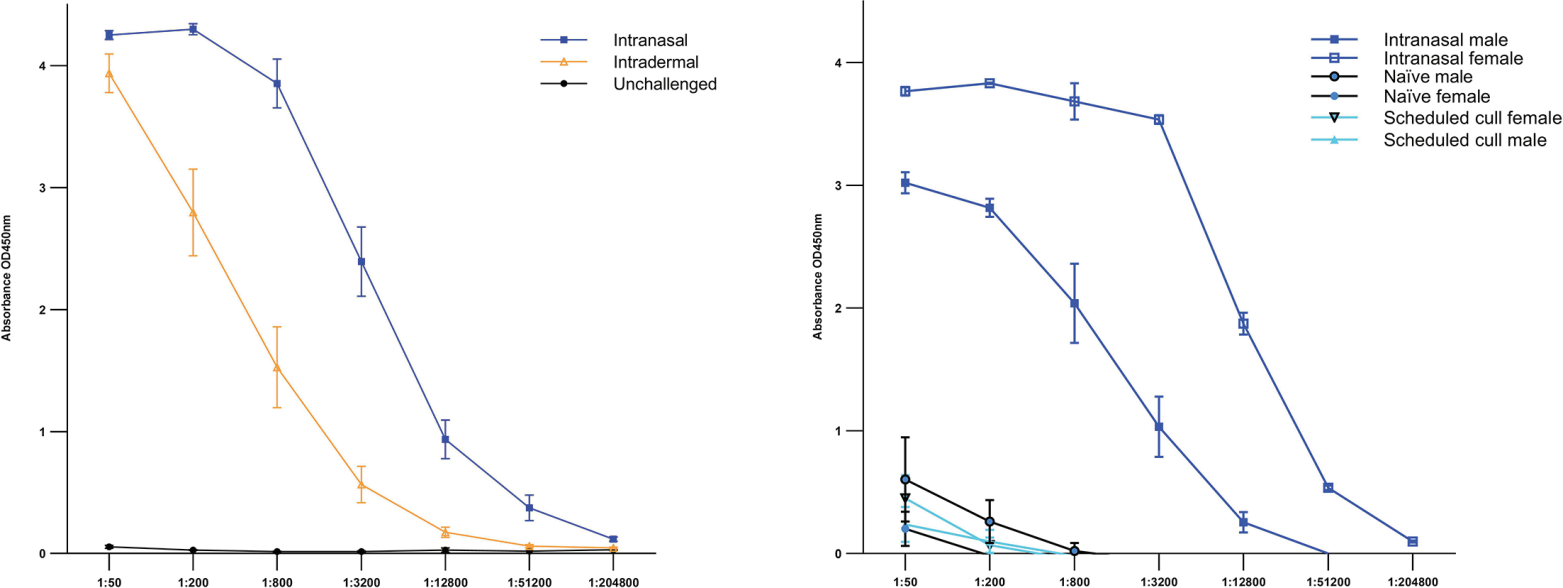
IgG responses to MPXV antigens from animals. ELISA absorbance values measured at 450 nm for both independent studies: susceptibility and transmission. (**a**) IgG responses to MPXV antigens from animals challenged intradermally and intranasally in the first study on susceptibility. IgG response to MPXV antigen from animals challenged by intranasal and intradermal routes observed a significant difference compared to control animals (*P*<0.05, Mann–Whitney U test). (**b**) IgG responses to MPXV antigens from intranasally challenged and naïve animals in the second study evaluating transmission. Intranasally challenged animals observed a significant increase in IgG response compared to their naïve cagemate (*P*<0.05, Mann–Whitney U test). Naïve animals developed a noticeable increase in IgG response at 21 dpi, similar to the response developed by challenged animals taken for scheduled cull at 6 dpi, with no significant difference (*P*>0.05, Mann–Whitney U test). Data has been normalized from background. Symbols show mean values with error bars denoting standard error.

Serum collected from all animals at the end of each study (21 dpi) was analysed by ELISA to measure the IgG response to MPXV antigens. Serum collected before challenge was used as a baseline measurement, with all animals being seronegative prior to challenge. In the first study assessing susceptibility, serum taken at 21 dpi from both the intranasally and intradermally challenged groups showed IgG responses to MPXV antigens, with a significant difference compared to the control group (*P*<0.05, Mann–Whitney U test) (Fig. 9a). The intranasally challenged group developed a higher IgG response compared to those challenged intradermally, although not significantly different (*P*>0.05, Mann–Whitney U test). In the second study assessing transmission, serum taken from challenged animals at 6 dpi and 21 dpi showed IgG antibody responses to MPXV antigens (Fig. 9b), further confirming results from the first susceptibility study. Sera taken from challenged animals in the scheduled cull group at 6 dpi observed an increase in IgG levels to MPXV antigen, and similar levels observed in sera from naïve cagemates who were housed with directly challenged animals up to 21 dpi. No significant difference was observed in IgG levels between the scheduled cull group at 6 dpi and naïve rats at 21 dpi (*P*>0.05, Mann–Whitney U test). The naïve cagemate animals had an observable increase in IgG levels to MPXV antigen, although at significantly lower levels to directly challenged animals with whom they co-housed up to 21 dpi (*P*<0.05, Mann–Whitney U test). A difference in antibody response levels was observed between sexes of challenged animals where challenged female rats developed a higher IgG response to MPXV antigens than males, although this did not reach statistical significance.

## Discussion

At the time of writing, MPXV infection in brown rats (*R. norvegicus*) has not been demonstrated. The aim of this study was to investigate brown rat susceptibility to MPXV and their transmission to see whether they could act as potential reservoir hosts to this viral pathogen. Two independent studies were conducted. In the first study for susceptibility, female brown rats were challenged with a recent strain of circulating MPXV (clade IIb), isolated from a positive patient case during the 2022 outbreak in the UK and used to evaluate infection. Challenge material used the highest concentration of virus stock (4×10^5^ TCID_50_) to evaluate brown rat susceptibility to mpox disease. The rats were challenged either intranasally or intradermally to imitate potential natural routes of infection of poxviruses in rodent hosts [6,11, 28, 29]. The second study for transmission replicated aspects from the first susceptibility study, where both male and female brown rats were challenged intranasally with clade IIb MPXV, alongside housing naïve rats to evaluate direct transmission. Clinical observations revealed no significant difference in weight or temperature readings for MPXV-challenged brown rats compared to negative controls over the course of the studies. Although it is common to see skin lesions as an indication of MPXV disease in humans [30] and established MPXV rodent animal models [29], no such manifestations were observed with the rats in both studies. Some animals in the transmission study were observed to have scabs, but these were inconsistent with mpox disease and observable alongside signs of overgrooming such as sores and hair loss localized on the head and were seen in animals from both challenged and unchallenged groups. Presenting scabs observed on rats were initially swabbed for RT-PCR, and skin samples from scabs were taken at necropsy. One skin sample showed one replicate negligible above the threshold, and all other samples were below the detectable threshold for MPXV DNA by RT-PCR, further confirming barbering behaviour in rats.

RT-PCR results of the first susceptibility study demonstrated the presence of viral MPXV DNA in lung tissue and throat swabs only after the intranasal challenge. Throat and rectal swabs were taken at 2–3-day intervals throughout the duration of the study, and tissues taken at necropsy were processed for PCR and histological analysis. We were unable to observe viral DNA in the rectal swabs; however, MPXV DNA was detectable in throat swabs from the intranasally challenged group from 2 to 10 dpi before being undetectable at 13 dpi. All throat swabs were negative on the day of challenge. At the time of necropsy, MPXV DNA was detected in three of the lung tissues taken from separate animals challenged via the intranasal route. Detectable MPXV DNA has been reported to be present in lung tissues from prairie dog animal models, associated with respiratory disease and thought to be a possible transmission route between rodents and from rodents to humans [31]. When attempting to grow a viable virus in Vero E6 cells, we did not observe CPE indicative of an infectious viable virus from the lung tissue samples. In contrast, viable virus CPE was observed in all the PCR-positive throat swabs taken at 4 dpi (*n*=5), demonstrating that brown rats are capable of maintaining a viable MPXV infection without the development of clinical signs of disease. Histopathological analysis was unable to identify the presence of virus in tissues using RNAscope assays, including those positive by PCR. This is likely due to the limit of detection where lower levels of DNA were only detectable by PCR. The second study assessing transmission produced replicable results with detectable MPXV DNA in the throat swabs from 2 to 10 dpi with peak infection at 6 dpi. MPXV DNA was detected in the intestine, kidney, brain, spleen and lung at necropsy of those taken at peak infection (6 dpi), with viable titres of 10^2^–10^4^ TCID_50_. No viral DNA was detected from tissues of infected animals at 21 dpi.

Despite no sign of disease or histopathological changes, both challenged groups in the first study demonstrated a high IgG antibody response to MPXV compared to similar studies in rodent species [2,29]. Sera from animals from the intranasally challenged route showed a greater IgG response to MPXV than those intradermally challenged. Similar studies show comparable results, where the Gambian pouched rat, responsible for the introduction of MPXV into the USA in 2003, exhibited sub-clinical infection in experimentally challenged intranasal groups, without presenting illness, although the virus could be detected in nasal and oral secretions [28]. Conversely, signs of clinical illness were observed in this species after challenge by the intradermal route, which is in contrast to our observations in brown rats. Whilst an IgG immune response to MPXV in the intradermally inoculated group was present, there was no viral DNA detectable by PCR in the swab or tissue samples collected. This could be due to intradermal challenge supporting localized primary infection and transport to the lymph nodes for early cellular responses, thus not reaching secondary viraemic release to tissues, resulting in subclinical infections in the absence of clinical disease [32,33]. Future work to characterize the role of mucosal immune responses between intranasal and intradermal challenge routes could be performed by evaluating the levels of IgA in serum samples and mucosal sites. The second independent study also produced replicable results from those intranasally challenged with MPXV. An IgG response was observed at the end of the study in the unchallenged cagemates, providing strong evidence of exposure to MPXV in this cohort. In this study, females produced a higher IgG response than males, and this, due to biological sex differences *in vivo*, is a contributing factor, as female rats have been shown to have a greater density of Toll-like receptors than their male counterparts [34]. Although sex differences were observed in ELISA assays, biological sex differences did not correlate with levels of viral load in swabs and tissues.

MPXV has been a cause of concern for public health since early May 2022, where many countries previously considered as non-endemic to MPXV faced a concerning outbreak of mpox disease at a pace and transmission rate that drove the WHO to declare the outbreak as a PHEIC [35]. Historically, MPXV is known to infect rodents, causing cyclic outbreaks in endemic countries [30], and because its natural reservoir is unknown, there is a need to investigate the susceptibility of infection in rodents which pose a serious public health risk.

Brown rats (*R. norvegicus*) have a worldwide distribution and frequently colonize habitats occupied by humans, consequently creating opportunities for the wider spread of zoonotic diseases [7]. It is known that a wide variety of rodents are susceptible to MPXV infection [36], with evidence from MPXV faecal and nasal secretions of infected rodents in previous studies [2,29]. Transmission of poxviruses from rats to humans has been previously described [16,17], but the potential for MPXV transmission between brown rats needs further investigation. Though aerosol transmission is less likely, the transmission of other viruses, such as hantaviruses, in brown rats has been shown to spread by wounding amongst rats, and the shedding of virus in urine, faeces and saliva, consequently infecting humans by the aerosolization of infected excretions [37]. *Orthohantaviruses*, alongside *Orthopoxviruses*, have been shown to be serologically present amongst wild rodents [38]. Seoul hantavirus (SEOV) is particularly prevalent amongst wild brown rats [39], and *Orthopoxviruses* such as CPXV have also been isolated from the *R. norvegicus* species [13,15]. There is a correlation between the geographical distribution of brown rats and the spread of hantaviruses across the world, where SEOV is found mainly in brown rats in South Asia but has also been discovered in brown rats from other countries across the world, including the UK [8,37]. Previous studies speculate that the faeces of SEOV-infected brown rats are a source of horizontal transmission; their findings showed that experimentally infected rats with low virus detection in the lung tissue reduce virus detection in faeces compared to wild naturally infected brown rats [40]. This correlation could explain our findings with only three animals showing detectable MPXV DNA in the lung tissue.

It is necessary to consider infectious excretions entering sewage systems. MPXV excretions in the faeces of rodents [29] could be a source of transmission into wastewater systems. Similarly, MPXV can also be detected in human faeces with the potential of excretions entering the wastewater systems [41], ultimately leading to the exposure of MPXV to brown rats living within sewers. *Orthopoxviruses* are known to be stable in the environment for long periods of time, and the presence of MPXV in wastewater systems has been detected at low DNA concentrations, although its stability and environmental transmission risk in wastewater systems are yet to be elucidated [42]. In this investigation, rectal swabs were periodically taken from rats throughout the susceptibility study for both intradermally and intranasally challenged animals and did not result in MPXV DNA being present by RT-PCR in excretory routes.

The susceptibility and transmission of MPXV in brown rats within this study were limited to the investigation of the West African strain (clade IIb) as a recent outbreak strain. Further investigations into brown rat susceptibility to MPXV and transmission with a Central African strain would be beneficial to understand the risk of exposure and spread. The Central African strain has been described as more virulent in clinical signs of disease in rodent animal models and known to downregulate host responses, specifically apoptosis in the host [43]. Additionally, transcriptional studies have shown that the Central African clade selectively silences the transcription of genes involved in host immunity by selectively blocking transcriptional responses of infected cells that are usually activated by TNF or IFNs [29,43, 44]. Further investigations into the intradermal infection site with RNAscope would be useful to determine if the virus disseminates beyond the infection site or whether the intradermal infection remains localized. Furthermore, exploration into the IgA antibody responses after both intranasal and intradermal challenge to study the role of mucosal barriers in MPXV-infected rats could be carried out, particularly as virus could be detected in the respiratory tract of the intranasally challenged group.

Our findings provide evidence that brown rats are asymptomatic to mpox disease, although the species is susceptible to sub-clinical infection, and therefore could act as a potential reservoir for future human infection. The intranasal route of infection gave a higher immune IgG response to MPXV compared to the intradermal route. Furthermore, MPXV DNA detected in infected rats was limited to rats inoculated via the intranasal route, with viable infectious virus isolated from the intranasal throat swabs providing potential of viral shedding from infected rats. Transmission of MPXV through infected brown rats has previously been unknown. Our results demonstrate that there is serological evidence that brown rats directly housed alongside challenged animals are exposed to MPXV at a level to mount a detectable IgG response, although no viral DNA was detected via RT-PCR except in the lung of one naïve animal that did not demonstrate any viable virus.

## Abbreviations

CPE: cytopathic effect
CPXV: cowpox virus
dpi: days post-infection
ELISA: enzyme-linked immunosorbent assay
H and E: haematoxylin and eosin
ISH: *in situ* hybridization
mpox: monkeypox
MPXV: mpox virus
PBS-T: PBS containing 0.05% Tween 20
PHEIC: public health emergency of international concern
RT-PCR: reverse transcription PCR
SEOV: Seoul hantavirus
WHO: World Health Organization

## References

[1] Xiang Y, White A (2022). Monkeypox virus emerges from the shadow of its more infamous cousin: family biology matters. Emerg Microbes Infect.

[2] Hutson CL, Nakazawa YJ, Self J, Olson VA, Regnery RL (2015). Laboratory investigations of African pouched rats (*Cricetomys gambianus*) as a potential reservoir host species for monkeypox virus. PLoS Negl Trop Dis.

[3] Hutson CL, Damon IK (2010). Monkeypox virus infections in small animal models for evaluation of anti-poxvirus agents. Viruses.

[4] Doshi RH, Guagliardo SAJ, Doty JB, Babeaux AD, Matheny A (2019). Epidemiologic and ecologic investigations of Monkeypox, likouala department, Republic of the Congo, 2017. Emerg Infect Dis.

[5] Hutson CL, Lee KN, Abel J, Carroll DS, Montgomery JM (2007). Monkeypox zoonotic associations: insights from laboratory evaluation of animals associated with the multi-state US outbreak. Am J Trop Med Hyg.

[6] Marennikova SS, Seluhina EM (1976). Susceptibility of some rodent species to monkeypox virus, and course of the infection. Bull World Health Organ.

[7] Puckett EE, Park J, Combs M, Blum MJ, Bryant JE (2016). Global population divergence and admixture of the brown rat (Rattus norvegicus. Proc Biol Sci.

[8] Strand TM, Lundkvist Å (2019). Rat-borne diseases at the horizon. A systematic review on infectious agents carried by rats in Europe 1995-2016. Infect Ecol Epidemiol.

[9] Kalthoff D, König P, Meyer H, Beer M, Hoffmann B (2011). Experimental cowpox virus infection in rats. Vet Microbiol.

[10] Weber S, Jeske K, Ulrich RG, Imholt C, Jacob J (2020). *In vivo* characterization of a bank vole-derived cowpox virus isolate in natural hosts and the rat model. Viruses.

[11] Breithaupt A, Kalthoff D, Deutskens F, König P, Hoffmann B (2012). Clinical course and pathology in rats (*Rattus norvegicus*) after experimental cowpox virus infection by percutaneous and intranasal application. Vet Pathol.

[12] Maiboroda AD (1982). Experimental infection of Norwegian rats (*Rattus norvegicus*) with ratpox virus. Acta Virol.

[13] Martina BEE, van Doornum G, Dorrestein GM, Niesters HGM, Stittelaar KJ (2006). Cowpox virus transmission from rats to monkeys, the Netherlands. Emerg Infect Dis.

[14] Kurth A, Wibbelt G, Gerber H-P, Petschaelis A, Pauli G (2008). Rat-to-elephant-to-human transmission of cowpox virus. Emerg Infect Dis.

[15] Wolfs TFW, Wagenaar JA, Niesters HGM, Osterhaus ADME (2002). Rat-to-human transmission of cowpox virus infection. Emerg Infect Dis.

[16] Campe H, Zimmermann P, Glos K, Bayer M, Bergemann H (2009). Cowpox virus transmission from pet rats to humans, Germany. Emerg Infect Dis.

[17] Vogel S, Sárdy M, Glos K, Korting HC, Ruzicka T (2012). The Munich outbreak of cutaneous cowpox infection: transmission by infected pet rats. Acta Derm Venereol.

[18] Baxby D, Bennett M (1997). Cowpox: a re-evaluation of the risks of human cowpox based on new epidemiological information. Arch Virol Suppl.

[19] Fenner F (1957). Studies in the epidemiology of infectious myxomatosis of rabbits. J Hyg.

[20] Thomas K, Tompkins DM, Sainsbury AW, Wood AR, Dalziel R (2003). A novel poxvirus lethal to red squirrels (*Sciurus vulgaris*). J Gen Virol.

[21] Damaso CR, Esposito JJ, Condit RC, Moussatché N (2000). An emergent poxvirus from humans and cattle in Rio de Janeiro State: cantagalo virus may derive from Brazilian smallpox vaccine. Virology.

[22] Leite JA, Drumond BP, Trindade GS, Lobato ZIP, da Fonseca FG (2005). Passatempo virus, a vaccinia virus strain, Brazil. *Emerg Infect Dis*.

[23] Venkatesan P (2022). Global monkeypox outbreak. Lancet Infect Dis.

[24] Zarocostas J (2022). Monkeypox PHEIC decision hoped to spur the world to act. The Lancet.

[25] Antinori A, Mazzotta V, Vita S, Carletti F, Tacconi D (2022). Epidemiological, clinical and virological characteristics of four cases of monkeypox support transmission through sexual contact, Italy, May 2022. Euro Surveill.

[26] Bragazzi NL, Kong JD, Mahroum N, Tsigalou C, Khamisy-Farah R (2023). Epidemiological trends and clinical features of the ongoing monkeypox epidemic: a preliminary pooled data analysis and literature review. J Med Virol.

[27] Li Y, Zhao H, Wilkins K, Hughes C, Damon IK (2010). Real-time PCR assays for the specific detection of monkeypox virus West African and Congo Basin strain DNA. J Virol Methods.

[28] Falendysz EA, Lopera JG, Lorenzsonn F, Salzer JS, Hutson CL (2015). Further assessment of monkeypox virus infection in *Cricetomys gambianus* using *in vivo* bioluminescent imaging. PLOS Negl Trop Dis.

[29] Hutson CL, Olson VA, Carroll DS, Abel JA, Hughes CM (2009). A prairie dog animal model of systemic orthopoxvirus disease using West African and Congo Basin strains of monkeypox virus. J Gen Virol.

[30] Domán M, Fehér E, Varga-Kugler R, Jakab F, Bányai K (2022). Animal models used in monkeypox research. Microorganisms.

[31] Guarner J, Johnson BJ, Paddock CD, Shieh W-J, Goldsmith CS (2004). Monkeypox transmission and pathogenesis in prairie dogs. *Emerg Infect Dis*.

[32] Hutson CL, Carroll DS, Gallardo-Romero N, Drew C, Zaki SR (2015). Comparison of monkeypox virus clade kinetics and pathology within the prairie dog animal model using a serial sacrifice study design. Biomed Res Int.

[33] Cho CT, Wenner HA (1973). Monkeypox virus. Bacteriol Rev.

[34] Scotland RS, Stables MJ, Madalli S, Watson P, Gilroy DW (2011). Sex differences in resident immune cell phenotype underlie more efficient acute inflammatory responses in female mice. Blood.

[35] World Health Organisation (2023). Mpox (monkeypox) outbreak: outbreak 2022 and subsequent developments. https://www.who.int/europe/emergencies/situations/monkeypox.

[36] Falendysz EA, Lopera JG, Rocke TE, Osorio JE (2023). Monkeypox virus in animals: current knowledge of viral transmission and pathogenesis in wild animal reservoirs and captive animal models. Viruses.

[37] Murphy EG, Williams NJ, Bennett M, Jennings D, Chantrey J (2019). Detection of Seoul virus in wild brown rats (*Rattus norvegicus*) from pig farms in Northern England. Vet Rec.

[38] Douglas KO, Cayol C, Forbes KM, Samuels TA, Vapalahti O (2021). Serological evidence of multiple zoonotic viral infections among wild rodents in Barbados. Pathogens.

[39] Clement J, LeDuc JW, Lloyd G, Reynes J-M, McElhinney L (2019). Wild rats, laboratory rats, pet rats: global Seoul hantavirus disease revisited. Viruses.

[40] Yasuda SP, Shimizu K, Koma T, Hoa NT, Le MQ (2021). Immunological responses to seoul orthohantavirus in experimentally and naturally infected brown rats (*Rattus norvegicus*). Viruses.

[41] de Jonge EF, Peterse CM, Koelewijn JM, van der Drift A-MR, van der Beek RFHJ (2022). The detection of monkeypox virus DNA in wastewater samples in the Netherlands. Sci Total Environ.

[42] Atoui A, Jourdain F, Mouly D, Cordevant C, Chesnot T (2023). A review on mpox (monkeypox) virus shedding in wastewater and its persistence evaluation in environmental samples. Case Stud Chem Environ Eng.

[43] Rubins KH, Hensley LE, Relman DA, Brown PO (2011). Stunned silence: gene expression programs in human cells infected with monkeypox or vaccinia virus. PLoS One.

[44] Kaler J, Hussain A, Flores G, Kheiri S, Desrosiers D (2022). Monkeypox: a comprehensive review of transmission, pathogenesis, and manifestation. Cureus.

