# Corrigendum: Susceptibility and transmission of mpox virus infection in brown rats (Rattus norvegicus)

**DOI:** 10.1099/jgv.0.002162

**Published:** 2025-10-10

**Authors:** Lucy Crossley, Stephen Findlay-Wilson, Linda Easterbrook, Emma Kennedy, Francisco J. Salguero, Kim Mackay, Victoria Graham, Susan Fotheringham, Stuart Dowall

**Affiliations:** 1 UK Health Security Agency (UKHSA), Porton Down, Salisbury, Wiltshire, SP4 0JG, UK

In the published version of this article, there are errors in the Methods section regarding the sources of recombinant proteins.

The Enzyme-linked immunosorbent assay section within the Methods of the manuscript states that recombinant MPXV antigens A35, B2, B6, VACV B5 and E8 were sourced from Native Antigen Company, UK.

However, the proteins were sourced from multiple companies. These are as follows: A35 (SinoBiological, China); B2 (Abbexa, UK); B6 and E8 (ProteoGenix, France); and VACV B5 (SinoBiological, China).

The authors apologise for any inconvenience caused.

